# A Low-Cost, Low-Skill Model for Efficient Breast Cancer Screening in Low Resource Rural Settings of a Developing Country

**DOI:** 10.7759/cureus.1571

**Published:** 2017-08-16

**Authors:** Sachin Khanduri, Mriganki Chaudhary, Tushar Sabharwal, Tarim Usmani, Aakshit Goyal, Shobha Khanduri, Saurav Bhagat, Fahimul Huda, Santosh Yadav, Gaurav Katyal

**Affiliations:** 1 Radiodiagnosis, Era's Lucknow Medical College and Hospital; 2 Pathology, Era's Lucknow Medical College and Hospital

**Keywords:** breast cancer, low resource rural settings, mammography, usg

## Abstract

Objective

To suggest a low-cost, easily-operable, non-invasive imaging modality for cancer detection in rural settings.

Method

A total of 212 cases with palpable breast masses aged 18 - 65 years were enrolled and underwent thorough clinical, mammographic, and ultrasonographic (USG) evaluation. Imaging findings were reported using the American College of Radiology (ACR) Breast Imaging Reporting and Data System (BI-RADS®) criteria. The findings were confirmed histopathologically. Data were analyzed using the Chi-square test.

Results

The malignancy rate was 35.8% (n = 76). On mammography, lesions size, margins, shape, calcification, and distorted arch/skin thickening were significantly associated with malignancy. On USG, the number of nodules, shape, margins, echotexture, posterior wall echo, through transmission changes, distorted arch/skin thickening, microlobulation, duct extension, and height/width ratio were significantly associated with malignancy. Independently, mammography and USG had a sensitivity of 78.1% and 80.3%, respectively, and a specificity of 83.3% and 89.0%, respectively. Using a positive result of either study as the criteria, the sensitivity was 97.4% and the specificity was 80.1%.

Conclusion

The combined use of mammography and USG provides high sensitivity and specificity, thus showing that a combination of two can be used as a screening tool for use in low resource rural settings.

## Introduction

With the increasing awareness regarding breast cancer and increased access to diagnostic modalities, the prevalence of breast cancer is increasing. The emergence of newer noninvasive diagnostic tools, such as magnetic resonance imaging, computed tomography, and positron emission tomography nuclear magnetic resonance (PET-NMR), has made the diagnosis of breast cancer somewhat easier. Early detection and treatment have been shown to be effective in order to combat the menace posed by breast cancer by increasing survival and resuming the quality of life of patients diagnosed at an early stage [1]. However, the picture is not as rosy as it appears to be. Unfortunately, the better diagnostic tools for breast cancer have failed to be passed on to all the strata of the society. The diagnostic set-ups, owing to their huge infrastructural costs, have become urban-centric, leading to deprivation of benefits to the rural masses.

India is a country, where despite rapid urbanization, more than two-thirds of the population (68.8%) still live in villages [2]. Economies of scale make it difficult to setup huge facilities in villages; as a result, the disparities in health care provision become visible. The screening facility for breast cancer detection in rural areas is jeopardized and, without exaggerating its status, has sometimes been termed as a non-existent reality [3]. Although there are reports that the odds of breast cancer are lower in the rural population as compared to the urban population in India [4], these studies are generally carried out in hospital populations and do not include the burden of undiagnosed cancer in the rural population; hence, their veracity remains to be proven. On the other hand, there are studies citing the barriers for early cancer detection amongst Indian rural women [5], as well as highlighting the lack of proper diagnostic services for late presentation of rural women.

With this backdrop, the present study was planned to target a screening facility for early diagnosis of breast cancer that is cost-efficient, does not requires highly skilled manpower, and is within the reach of the rural population. Mammography and ultrasonography are two relatively economical imaging modalities that do not require many infrastructural costs as well as skills; hence, the present study has evaluated their independent/combined usefulness for the stated objective.

## Materials and methods

The study was carried out at Era’s Lucknow Medical College and Hospital (ELMCH), Lucknow, India, a facility catering mainly to the semiurban and rural Lucknow population, between January 2015 to June 2016. A total of 212 women aged 18 - 65 years with palpable breast masses suspected of breast cancer were included in the assessment. Pregnant and lactating women, as well as those with pacemakers and/or metallic clips, were excluded from the assessment. The project was approved by the Institutional Ethical Committee of ELMCH (#ELMC/EC/R_Cell/2014/220). Informed consent was obtained from all the participants.

Demographic information and details regarding the side involved, pain, discharge, retraction, and heaviness of breasts were noted.

A thorough physical examination was performed that focused on the mobility of the breast mass(es), warmth on touch/redness, pain on palpation, hardness, and any other relevant physical characteristics. After the clinical examination was over, the patients underwent mammographic assessment, which was performed using a stand-type Allengers VENUS (Allengers Medical Systems, Ltd., Chandigarh, India) mammography machine. When the lesion was located, the side of involvement, the lesion size, pattern of margins of the lesion, shape of the lesion, density of the mass, presence of calcifications, and other associated features, such as the presence of a dilated duct, distorted arch, nipple retraction, skin thickening, etc., were noted.

All the patients then underwent ultrasonographic examination with the help of a GE Voluson P8 (GE Healthcare, Little Chalfont, United Kingdom) ultrasonography machine, using a high-frequency 7-12 MHz linear electronic array transducer. During the ultrasonography (USG) examination, the number of nodules, quadrant of breast involved, side of involvement, size of the nodule(s), shape of the lesion, margins, echogenicity, echotexture, posterior wall echo pattern, through-transmission changes, and associated features, such as presence of dilated duct, distorted arch, nipple retraction, and skin thickening, were noticed.

The radiological/imaging findings were reported using the American College of Radiology (ACR) Breast Imaging-Reporting and Data System (BI-RADS) criteria (Table 1).

**Table 1:** Criteria for Reporting (BI-RADS) This table represents assessment categories on the basis of mammographic and ultrasonographic examinations, according to the American College of Radiology (ACR) Breast Imaging-Reporting and Data System (BI-RADS). SN: serial number Source: D’Orsi CJ, Sickles EA, Mendelson EB, Morris EA, et al.: ACR BI-RADS® Atlas, Breast Imaging-Reporting and Data System. Reston, VA, American College of Radiology; 2013

SN	Diagnosis	Corresponding BI-RADS	Features
1.	Negative / Needs further evaluation	BI-RADS 0/I	Lesion not detectable
2.	Benign	BI-RADS II	Detectable, well-defined margins, oval to round shape, intermediate/high density, popcorn/coarse/no calcification, no associated features.
3.	Probably Benign	BI-RADS III	Most of the features corresponding to the benign category. Only a few overlapping features
4.	Probably Malignant	BI-RADS IV	One or more of following: ill-defined, irregular/lobulated/microlobulated, high-density, presence of associated features
5.	Malignant	BI-RADS V	Clearly visible two or more of features with spiculated margins, microcalcifications, distorted architecture, nipple retraction

For the purpose of the present study, BI-RADS IV/V categories were considered as malignant and all the lower BI-RADS grades were considered as benign.

Histopathology

Histopathological specimens were obtained by the surgeons at ELMCH and were sent to the Pathology Department for further assessment. The histopathological diagnosis was considered final. Correlation of the histopathological and radiological/imaging results was done. For combined assessment of mammography, plus USG, a positive result of either study was taken as the criteria.

Data collected were subjected to statistical analysis.

Data analysis

Data were analyzed using the Statistical Package for Social Sciences (SPSS) version 20.0 (IBM SPSS Statistics, Armonk, NY). Chi-square tests were used to compare the data. A p-value (probability of chance error) less than 0.05 indicated a significant association.

## Results

Age of the patients ranged from 18 to 64 years with the majority being < 40 years of age (73.1%). The mean age of patients was 37.8 + 10.41 years. The left side was more commonly involved (n = 106; 50%), followed by the right side (n = 95; 45.3%). Bilateral involvement was observed in 10 (4.7%). Pain (17%), discharge from the nipple (25.9%), nipple retraction (16.5%), and heaviness (13.7%) were some of the common characteristic features (Table 2).

**Table 2:** Demographic and Clinical Profile of Patients Enrolled in the Study This table represents characterization of demographic and clinical profile of the patients enrolled in the study and their corresponding statistical values. SN: serial number; SD: standard deviation

SN	Characteristic	Statistic
1.	Mean Age ± SD (range) in years	37.8 ± 10.41 (18-64)
	< 40 yrs	155 (73.1%)
	> 40 yrs	57 (26.9%)
2.	Side involved	
	Left	106 (50.0%)
	Right	96 (45.3%)
	Bilateral	10 (4.7%)
3.	Pain	36 (17.0%)
4.	Discharge	55 (25.9%)
5.	Retraction	35 (16.5%)
6.	Heaviness	29 (13.7%)

**Figure 1:**
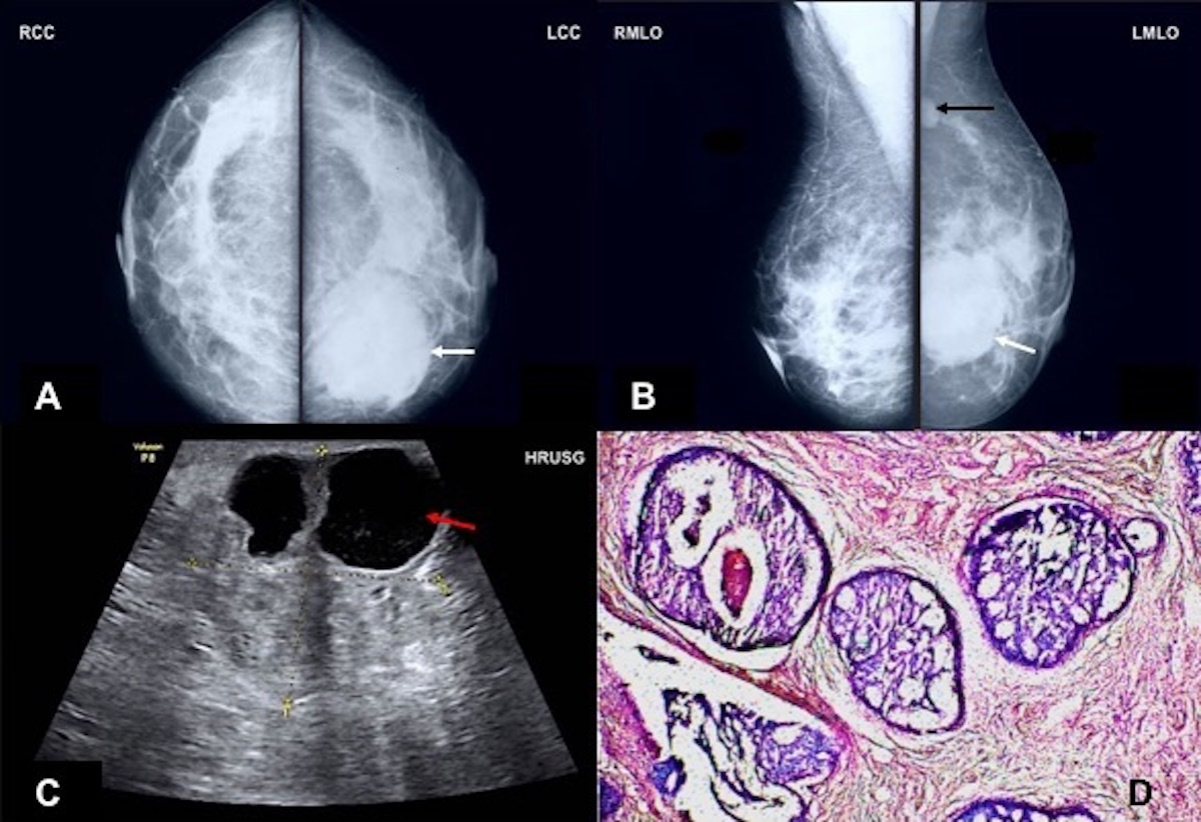
A 44-year-old female patient with intraductal carcinoma variant A) Mammographic craniocaudal and B) mediolateral oblique views of the left breast show a high-density mass in inferomedial quadrant (white arrow), along with ipsilateral axillary lymphadenopathy (black arrow). C) High-resolution ultrasonography image of the left breast shows a heterogeneously hyperechoic mass with anechoic component with fine internal echoes at 5 o'clock position. D) Photomicrograph of this section shows glands in cribriform pattern lined by atypical pleomorphic cells with hyperchromatic nuclei and high nuclear:cytoplasmic ratio (hematoxylin and eosin stain 10X).

**Figure 2:**
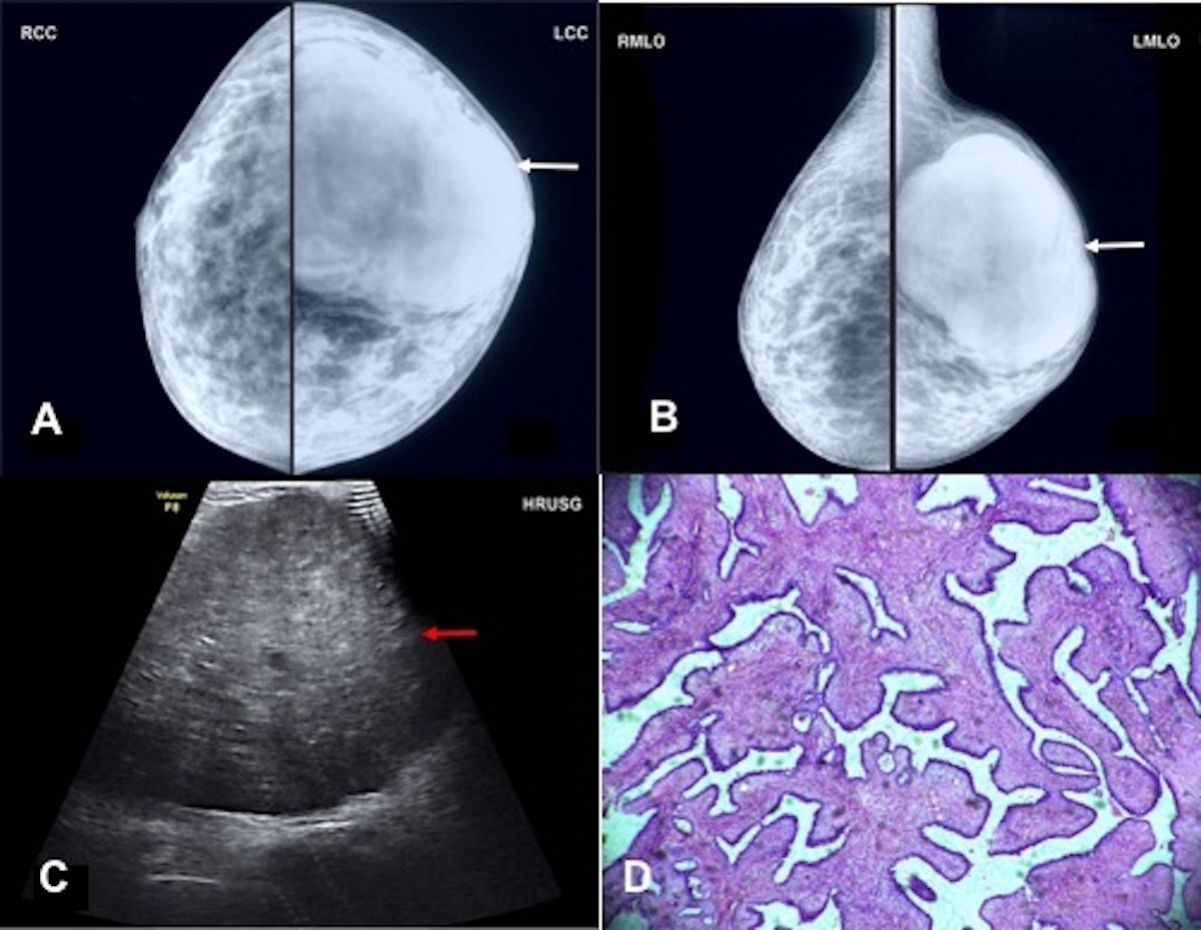
A 35-year-old female patient of phyllodes tumor A) Mammographic craniocaudal and B) mediolateral oblique views of left breast shows a large, lobulated high density mass occupying the entire upper quadrant (white arrow). C) High-resolution ultrasonography image of left breast shows a large, lobulated heterogeneously hypoechoic mass occupying the entire right breast extending from 8 o'clock to 4 o'clock position (red arrow). D) Photomicrograph of this section shows marked stromal proliferation with stroma bulging into the glandular lining forming a leaf like architecture (hematoxylin and eosin 4X).

Histopathologically, a total of 76 (35.8%) cases proved to be malignant. Fibroadenoma (benign) was the most common finding (n = 88; 41.5%), followed by intraductal carcinoma-Variant (n = 43; 20.3%) (Figure 1), and intraductal carcinoma (well-differentiated) (n = 24; 11.3%). A total of 10 (4.7%) were diagnosed as intraductal papilloma, nine (4.2%) as invasive lobular carcinoma, and eight (3.8%) as a breast abscess. There were seven (3.3%) cases of a complex cyst and five (2.4%) cases each of galactocele and fibrocystic breast disease, respectively. Benign intramammary nodes were diagnosed in four (1.9%) cases. Three (1.4%) cases each were diagnosed as fibroadenolipoma, phyllodes (Figure 2), and tubercular breast abscess, respectively (Table 3).

**Table 3:** Histopathological Diagnosis This table represents characterization of the histopathological diagnosis and their corresponding numerical and percentile values. SN: serial number; No: number; IDC-V: intraductal carcinoma-variant; IDC-WD: intraductal carcinoma-well-differentiated

SN	Finding	No.	Percentage (%)
1.	Benign intramammary node (Benign)	4	1.9
2.	Breast abscess (Benign)	8	3.8
3.	Fibroadenolipoma (Benign)	3	1.4
4.	Fibroadenoma (Benign)	88	41.5
5.	Fibrocystic breast disease (Benign)	5	2.4
6.	Galactocele (Benign)	5	2.4
7.	Intraductal papilloma (Benign)	10	4.7
8.	Phyllodes (Benign)	3	1.4
9.	Complex cyst (Benign)	7	3.3
10.	Tubercular breast abscess (Benign)	3	1.4
11.	IDC-V (Malignant)	43	20.3
12.	IDC-WD (Malignant)	24	11.3
13.	Invasive Lobular Carcinoma (Malignant)	9	4.2

Mammographically, lesions could be evaluated in 199 (93.9%). In 13 cases, the lesion could not be detected - owing to unilateral asymmetric focal increased density in 10 cases and asymmetric increased density in the remaining three cases. Hence, the mammographic evaluation was limited to 199 cases only. Unilateral involvement was more common (n = 190; 95.5%). Only nine (4.5%) cases had bilateral involvement. The left side was more commonly involved (n = 102; 51.3%) than the right side (44.2%). Statistically, no significant association between the side of involvement and the final diagnosis was seen (p = 0.885). The majority of lesions ranged in size between 2 - 5 cm (n = 120; 51.3%). There were 60 (30.2%) lesions that measured < 2 cm and 19 (9.5%) measured above 5 cm. In the malignant group, the proportion of lesions > 2 cm size was significantly higher (87.7%) as compared to that in the benign group (59.5%) (p < 0.001). The majority of tumors with well-defined margins were benign (n = 82; 65.1%) as compared to only six (8.2%) of the malignant cases. Statistically, this was a significant difference between the two diagnostic entities (p < 0.001). The ill-defined shape of a lesion was more common in malignant cases (n = 48; 65.8%); however, the well-defined shape of a lesion was more common in benign cases (65.1%). Statistically, this difference was significant (p < 0.001). The majority of malignant (65.8%) and benign (51.6%) cases had high density. Statistically, there was no significant difference in the density pattern (p = 0.113). The presence of microcalcification was a characteristic finding of malignant lesions. However, it was present in only 17 (23.3%) of malignant cases as compared to only one (0.8%) of benign cases, while popcorn and coarse calcifications were the most common calcification features in benign as compared to malignant lesions. Statistically, whenever calcification was present, it showed a significant difference in presentation between the two groups (p < 0.001). Most of the benign cases did not have any other associated feature. In benign cases, the associated features were seen as dilated duct and distorted architecture in 3.2% and 1.6% cases, respectively. However, the malignant group had a number of associated features on mammography; as many as 28 (38.4%) cases had distorted architecture, three cases had nipple retraction with distorted arch, and two cases had skin thickening. Thus, associated features, such as distorted architecture, nipple retraction, and skin thickening, were characteristic of the malignant group. Statistically, there was a significant difference between the two groups with respect to associated features (p = 0.001). On the basis of above characteristics, out of 199 cases analyzed mammographically, 78 (39.2%) were diagnosed as malignant and the remaining 121 (60.8%) were diagnosed as benign (Table 4).

**Table 4:** Mammographic Findings and Their Correlation with Histopathological Findings (n = 199)* This table represents categorization of mammographic, as well as histopathological, findings for malignant and benign lesions in both numerical and percentile forms, along with their statistical significance SN: serial number; n: total number of cases in that category; X^2^: Chi-square; p: probability of chance error *Mammography could not detect 13 lesions

SN	Finding	Total (n=199)		Histopathological Finding				Statistical significance	
				Malignant (n=73)		Benign (n=126)			
		No.	%	No.	%	No.	%	X^2^	p
1.	Detectable lesion*	199/212	93.9	73/76	96.1	126/136	92.6	0.982	0.322
2.	Side involved								
	Right	88	44.2	32	43.8	56	44.4	0.245	0.885
	Left	102	51.3	37	50.7	65	51.6		
	Bilateral	9	4.5	4	5.5	5	4.0		
3.	Size								
	< 2 cm	60	30.2	9	12.3	51	40.5	19.4	< 0.001
	2 - 5 cm	120	60.3	58	79.5	62	49.2		
	> 5 cm	19	9.5	6	8.2	13	10.3		
4.	Margins								
	Ill-defined	31	15.6	13	17.8	18	14.3	88.2	< 0.001
	Lobulated	7	3.5	3	4.1	4	3.2		
	Microlobulated	9	4.5	6	8.2	3	2.4		
	Oval	4	2.0	1	1.4	3	2.4		
	Partially well-defined	21	10.6	11	15.1	13	10.3		
	Round	5	2.5	3	4.1	2	1.6		
	Spiculated	31	15.6	30	41.1	1	0.8		
	Well-defined	88	44.2	6	8.2	82	65.1		
5.	Shape								
	Ill-defined	61	30.7	48	65.8	13	10.3	71.1	< 0.001
	Lobulated	38	19.1	12	16.4	26	20.6		
	Oval/Round	97	48.7	13	17.8	84	66.7		
	Well-defined	3	1.5	0	0.0	3	2.4		
6.	Density								
	High	112	56.3	47	64.4	65	51.6	4.36	0.113
	Intermediate	76	38.2	24	32.9	52	41.3		
	Low	11	5.5	2	2.7	9	7.1		
7.	Calcification								
	Absent	157	78.9	56	76.7	101	80.2	37.2	< 0.001
	Coarse	10	5.0	0	0.0	10	7.9		
	Micro	18	9.0	17	23.3	1	0.8		
	Popcorn	14	7.0	0	0	10	7.9		
8.	Associated Features								
	Absent	160	80.4	40	54.8	120	95.2	61.8	< 0.001
	Dilated duct	4	2.0	0	0.0	4	3.2		
	Distorted arch	30	15.1	28	38.4	2	1.6		
	Distorted arch, nipple retraction	3	1.5	3	4.1	0	0.0		
	Skin thickening	2	1.0	2	2.7	0	0.0		
9.	Mammographic Diagnosis								
	Malignant	78	39.2	57	78.1	21	16.7	73.2	< 0.001
	Benign	121	60.8	16	21.9	105	83.3		

On USG, the majority of cases in both the groups had only one nodule. There were 11 malignant cases and three benign cases who had two nodules. More than two nodules were seen in 13.2% of malignant cases and none of the benign cases. Statistically, the difference in the number of nodules was significant (p < 0.001). The upper quadrant was more commonly involved in malignant cases (57.9%) as well as in benign cases (51.5%); however, the difference between the two groups was not significant statistically (p = 0.788). Most of the cases had unilateral involvement. The left side was more commonly involved than the right side. Bilateral involvement was seen in 10 (4.7%) cases only. Statistically, there was no significant difference between the two groups with respect to the side involved (p = 0.959). The size of the lesion was 2 cm or more in most of the cases (67.5%). The proportion of those with a size > 2 cm was significantly higher in the malignant group (84.2%) as compared to the benign group (58.1%) (p < 0.00). The majority of the malignant cases had an irregular/ill-defined shape (64.8%), whereas the majority of benign cases (72.8%) had an oval/round or well-defined shape. Statistically, this difference between the two groups was significant (p < 0.001). Ill-defined margins were seen in the majority of malignant cases (55.3%), whereas only 20 (14.7%) benign cases had ill-defined margins; the majority of benign cases had well-defined margins (70.6%) as compared to only four (5.3%) of the malignant cases. Statistically, there was a significant difference between the two groups with respect to the pattern of margins (p < 0.001). Irrespective of the diagnosis, the majority of cases in both groups had hypoechoic patterns. Isoechoic patterns were seen in nine (4.2%) cases and heteroechoic patterns in only 16 (7.5%) cases (p = 0.664). Heterogenous echotexture was more common in malignant cases (55.3%) as compared to only 20 (14.7%) of the benign cases. Statistically, the difference in echotexture between the two groups was significant (p < 0.001). In both groups, a posterior wall echo pattern was most common; however, the proportion of cases having a weak posterior wall echo pattern was significantly higher in the malignant group (18.4%) as compared to benign group (3.7%) (p < 0.001). No change was seen in through-transmission in the majority of cases of both the groups; however, an enhancement was observed in a significantly higher proportion of benign cases (43.4%) as compared to none in the malignant cases (6.6%) (p < 0.001). As such, the presence of associated features was significantly higher in malignant cases (n = 36; 47.4%) as compared to benign cases (8.1%) (p < 0.001). Microlobulations and features, such as duct extension and nodular size taller than wider, were more common in the malignant group as compared to the benign group, thus showing a significant difference between the two groups (p < 0.001). On the basis of the above characteristics, the USG diagnosis was malignant in 76 (35.8%) cases and benign in the remaining 136 (64.2%) cases (Table 5).

**Table 5:** Ultrasonographic Findings and Their Correlation with Histopathological Findings This table represents characterization and correlation of ultrasonographic and histopathological findings for malignant and benign lesions in both numerical and percentile forms, along with their statistical significance SN: serial number; No.: numbers; X^2^: Chi-square; p: probability of chance error; USG: ultrasonographic

SN	Finding	Total		Histopathological finding				Statistical significance	
				Malignant (n = 76)		Benign (n = 136)			
		No.	%	No.	%	No.	%	x^2^	p
1.	No. of nodules								
	1	188	88.7	55	72.4	133	97.8	32.6	< 0.001
	2	14	6.6	11	14.5	3	2.2		
	> 2	10	4.7	10	13.2	0	0.0		
2.	Quadrant								
	Upper	114	53.8	44	57.9	70	51.5	1.05	0.788
	Lower	55	25.9	19	25.0	36	26.5		
	Multiple	22	10.4	7	9.2	15	11.0		
	Retroareolar	21	9.9	6	7.9	15	11.0		
2.	Side involved								
	Right	96	45.3	34	44.7	62	45.6	0.827	0.959
	Left	106	50.0	38	50.0	68	50.0		
	Bilateral	10	4.7	4	5.3	6	4.4		
3.	Size								
	< 2 cm	69	32.5	12	15.8	57	41.9	19.8	< 0.001
	2-5 cm	122	57.5	59	77.6	63	46.3		
	> 5 cm	21	9.9	5	6.6	16	11.8		
4.	Shape								
	Irregular/Ill-defined	59	27.8	49	64.5	10	7.4	87.4	< 0.001
	Lobulated	42	19.8	15	19.7	27	19.9		
	Oval/Round/Well-defined	111	52.4	12	15.8	99	72.8		
5.	Margins								
	Ill-defined	62	29.2	42	55.3	20	14.7	91.5	< 0.001
	Lobulated	3	1.4	2	2.6	1	0.7		
	Partially ill-defined	35	16.5	17	22.4	18	13.2		
	Spiculated	12	5.7	11	14.5	1	0.7		
	Well-defined	100	47.2	4	5.3	96	70.6		
6.	Echogenicity								
	Heteroechoeic	16	7.5	7	9.2	9	6.6	0.820	0.664
	Hypoechoiec	187	88.2	65	85.5	122	89.7		
	Isoechoeic	9	4.2	4	5.3	5	3.7		
7.	Echotexture								
	Heterogenous	82	38.7	52	68.4	30	22.1	44.2	< 0.001
	Homogenous	130	61.3	24	31.6	106	77.9		
8.	Posterior Wall Echo								
	Strong	35	16.5	7	9.2	28	20.6	15.9	< 0.001
	Intermediate	160	75.5	55	72.4	105	77.2		
	Weak	17	8.0	14	18.4	5	3.7		
9.	Through-Transmission								
	Attenuation	13	6.1	4	5.3	9	6.6	35.1	< 0.001
	Enhancement	51	24.1	1	1.3	50	36.8		
	No change	148	69.8	71	93.4	77	56.6		
10.	Associated Features								
	Absent	165	77.8	40	52.6	125	91.9	72.3	< 0.001
	Dilated duct	9	4.2	0	0.0	9	6.6		
	Distorted arch	28	13.2	27	35.5	1	0.7		
	Distorted arch, nipple retraction	3	1.4	3	3.9	0	0.0		
	Skin thickening	7	3.3	6	7.9	1	0.7		
11.	Microlobulations	70	33.0	57	75.0	13	9.6	94.4	< 0.001
12.	Duct extension	62	29.2	53	69.7	9	6.6	113.0	< 0.001
13.	Taller than wider	71	33.5	54	71.1	17	12.5	93.7	< 0.001
14.	USG Diagnosis								
	Malignant	76	35.8	61	80.3	15	11.0	102.0	< 0.001
	Benign	136	64.2	15	19.7	121	89.0		

We evaluated the diagnostic efficacy of mammography, USG, and the combination of mammography, plus USG. Using mammography (n = 199), a total of 57 cases were true positive, 21 were false positive, 16 were false negative, and 105 were true negative; correspondingly sensitivity, specificity, positive predictive value (PPV), and negative predictive values (NPV) were 78.1%, 83.3%, 73.1%, and 86.8%, respectively. Mammography had an accuracy of 81.4%. Using USG, a total of 61 cases were true positive, 15 were false positive, 15 were false negative, and 121 were true negative; correspondingly sensitivity, specificity, positive predictive values, and negative predictive values were 80.3%, 89.0%, 80.3%, and 89.0%, respectively. USG had an accuracy of 85.8%. Using the combination of mammography, plus USG (either positive criteria), a total of 74 cases were true positive, 27 were false positive, two were false negative, and 109 were true negative; correspondingly sensitivity, specificity, positive predictive value, and negative predictive values were 97.4%, 80.1%, 73.3%, and 98.2%, respectively. The combination had an accuracy of 86.3% (Table 6).

**Table 6:** Diagnostic Efficacy of Mammography, USG, and Combination of Mammography + USG Against Histopathological Findings This table represents statistical characterization referring to diagnostic efficacy of mammography, ultrasonography, and their combination against histopathological findings SN: serial number; TP: true positive; FP: false positive; PPV: positive predictive value; NPV: negative predictive value; Sens: sensitivity; Spec: specificity

SN	Variable	TP	FP	FN	TN	Sens	Spec	PPV	NPV	Accuracy
1.	Mammography alone	57	21	16	105	78.1	83.3	73.1	86.8	81.4
2.	USG alone	61	5	15	121	80.3	89.0	80.3	89.0	85.8
3.	Mammography + USG	74	27	2	109	97.4	80.1	73.3	98.2	86.3

## Discussion

In the present study, the majority of patients were < 40 years of age. As far as the range of age is concerned, it is disappointing to see that people at younger ages are being affected by breast cancer; however, this is not an unusual finding. In India, unlike the West, an increasingly higher proportion of women in younger age groups (< 45 years) have been shown to be affected by breast cancer [6]. Even in the West, studies have reported breast cancer patients at a younger age. In different studies, the age of patients has ranged from 21 to 77 years with the mean age around 42 to 45 years [7-9].

In the present study, the histologically proven malignancy rate was 35.7%. The malignancy rate has been reported to vary substantially from 6.2% to 53.5% in different studies [7-8, 10-13]. Chakraborti, et al. (2005), in a study similar to ours, found the malignancy rate to be 30% [14]. The variability in the malignancy rate is dependent on the clinical status and profile of the population being screened and shows a substantial variability in different studies.

Histopathologically, the present study found infiltrative ductal carcinoma to be the major presentation, followed by invasive lobular carcinoma in the remaining cases. Similar to our findings, Wasif, et al. (2009) reported infiltrating ductal cancer in 83.9% cases and had invasive lobular cancer in 6.0% cases [15]. Various epidemiological studies also place infiltrating ductal carcinoma to be the most common type, comprising nearly 75-80% of breast carcinoma types [16-17]. The findings of the present study also endorsed these observations.

In the present study, mammography was 78.1% sensitive and 83.3% specific for malignancy; it had a PPV of 73.1%, NPV of 86.8%, and accuracy of 81.4% for malignant tumors. Mammography is the primary imaging modality for breast cancer screening and diagnosis, and it is generally more sensitive. Its sensitivity is reported to be above 80% [7]. However, in the present study, it was found to be more specific than sensitive. The highly specific role of mammography is reported under certain conditions, such as in carriers of breast cancer (BRCA) mutations [18]. It is difficult to state whether our study subjects had BRCA mutations or not since BRCA evaluation was not part of our study. The reason for the higher specificity of mammography in the present study could be the use of the digital mammography technique. In the present study, mammography was found to have an 81.4% accuracy. However, in the study of Hlawatsch, et al. (2002), mammography proved to have an accuracy of only 48% [12]. Similar to our study, Chakraborti, et al. (2005) have also reported a lower sensitivity (65%) of mammography for non-palpable lesions [11]. In the present study, we took only palpable masses into consideration and this could be the reason for the lower sensitivity in the present study. However, there are studies in recent literature that have reported the sensitivity of mammography to be only 57.1% in a high-risk population undergoing mammographic screening [9]. In another recent study, although the sensitivity was reported to be 88.5%, the specificity was limited only to 57.9% [19]. Kuhl, et al. (2005), in a different set of the population of asymptomatic women, also found reported the sensitivity of mammography to range from 25% to 33% among low-risk and high-risk patients [20]; however, they reported a high specificity of mammography (96.8%). In the present study, too, symptomatic manifestation was low; pain (17%), discharge (25.9%), retraction (16.5%), and heaviness (13.7%) were less reported symptoms at presentation. The specificity rate for mammography in the present study is similar to that reported in 2013 by Taori, et al. [21].

Among different mammographic findings indicative of malignancy, size > 2 cm, ill defined or irregular margins, ill-defined shape, microcalcifications, and distorted arch were some of the significant distinguishing features. The size of the tumor is considered to be a predictor of malignancy. In a study comparing high-resolution ultrasonography (HR-USG) and mammography, Chan, et al. (2008) found that size assessment using mammography did not show a significant difference from HR-USG, thus suggesting that mammography might provide a good clue about malignancy using the size of the lesion as the criteria [22]. In another study, Wasif, et al. (2009) showed that mammography, with respect to size, is more sensitive [15]. In the present study, mammography showed the role of margin patterns in the diagnosis of malignancy and highlighted that well-defined margins were more common in benign masses as compared to malignant masses. It is a finding in accordance with the literature [23]. Similar to our study, irregular mass shape and the presence of calcification have been shown to be characteristic mammographic features of malignancy [24-25]. Thus, the present study showed that, if a single feature criterion like an ill-defined shape is taken into consideration, the sensitivity of mammography could be low at 65%, but its specificity could be increased to 89.7%. However, keeping in view the purpose of mammography to provide a more sensitive outcome, we employed the use of multiple factor criteria, and hence, both sensitivity and specificity could be brought into a reasonable range.

In the present study, USG characteristics, such as the number of lesions, shape, margins, echotexture, posterior wall echo, through-transmission changes, and the presence of associated features, e.g., microlobulations and duct extension, provided useful clues for diagnosis. These findings are in agreement with the observations made by Stavros, et al. (1995) who reported that echogenic patterns, shape, and lobulations are sonographic features that help in distinguishing malignant from benign lesions [10]. In a recent study, heterogeneity, partially indistinct margins, and microlobulations were shown to be useful in assigning a higher BI-RADS grade to a suspicious breast lesion [26]. In the present study, using the same criteria, we were able to distinguish the majority of cases finally diagnosed as malignant on sonography.

In the present study, we intended to use the mammography with or without USG as a screening modality for breast cancer detection in a low-resource rural setting and failed to get a single criterion with a high sensitivity as well as specificity. Hence, we further investigated whether a combination of these two modalities could further enhance the sensitivity and, as such, make the preliminary diagnosis earlier at the primary care giving facility itself. On combining the two modalities and taking either positive as the criteria, we were able to achieve a sensitivity of 97.4% and specificity of 80.1% while, at the same time, having a positive and negative predictive value of 73.3% and 98.2%, respectively. The overall accuracy of the model was 86.3%. These findings suggested that when a combination criterion is used at the primary rural settings itself, then there are only 3 out of 100 chances of missing a breast cancer, whereas an additional burden of carrying further assessment is around 26.7%. Compared to this, when no screening is done, the burden of additional carriage for advanced assessment is as high as 64.1%, which implies that urban facilities will be squeezed with an additional burden of nearly 37.4% on their resources if no such rural settings are made available.

The high sensitivity of the combined assessment is the hallmark of this combinatorial criterion specially designed for rural settings where advanced diagnostic facilities and histopathological assessment are not possible. Similar to our study, several other studies have reported that the diagnostic accuracy of mammography and HR-USG increases when used in combination [11]. In the present study, the combination criteria had a higher sensitivity than both the techniques independently. However, Hou, et al. (2002) found using combined criteria to be less sensitive than USG or mammography alone [27]. In the present study, we took either positive as the basis of categorization in combined criteria and were able to obtain a high sensitivity with a primary objective to enhance the sensitivity of the combinatorial model. It is essential that relaxed criteria be chosen instead of strict criteria to get a more sensitive screening modality. In the present study, we were quite clear in our approach to develop a criterion that had a higher sensitivity, and finally, using an either positive criteria achieved a high sensitivity without any substantial loss of specificity. This could have been possible because, for USG and mammography diagnoses, we made diagnoses based on a multitude of factors rather than a single factor and thus, made it more specific at that level itself. An increase in sensitivity of mammography in combination with USG has also been reported by some other authors [12-13, 28]. Using relaxed criteria, as used in this study, resulted in an absolute sensitivity in a study by Prasad and Houserkova (2007) [13]. Using a strict criterion, Taori, et al. (2013) reported the specificity of the combination to be as high as 97% [26]. Other authors have also supported the use of combined criteria to show an increase in diagnostic efficacy, as observed in the present study [29-30].

## Conclusions

The findings in the present study showed that for a rural setup, mammography with USG is a suitable diagnostic modality with an adequate sensitivity and a reasonable specificity. We must focus on sensitivity using relaxed criteria in order to rule out the exclusion of any true positive case. Screening might help in reducing the additional burden at an urban centre as well as the woes of rural folks. This will help in making an early diagnosis, followed by an initial treatment. Further studies on other predictive features of USG/mammography are recommended with a larger sample size to validate the rationality of results of the present study.
